# Utilizing Patient Reported Outcome Measures (PROMs) in ambulatory oncology in Alberta: Digital reporting at the micro, meso and macro level

**DOI:** 10.1186/s41687-021-00373-3

**Published:** 2021-10-12

**Authors:** Linda Watson, Andrea Delure, Siwei Qi, Claire Link, Lindsi Chmielewski, Éclair Photitai, Louise Smith

**Affiliations:** 1 Cancer Research and Analytics, Cancer Care Alberta, Alberta Health Services, Holy Cross Site, Box ACB, 2210-2nd ST SW, Calgary, AB T2S 3C3 Canada; 2 Faculty of Nursing, University of Calgary, Calgary, AB Canada

**Keywords:** Patient Reported Outcome Measures, PROMs, PROs, Cancer, Symptom complexity level, Complexity algorithm, PRO dashboards, Symptom management, Routine, Dashboards, Ambulatory oncology, Micro, Meso, Macro, Aggregate, Person centred care, Personalized care

## Abstract

Cancer patients experience numerous distressing symptoms and concerns across the course of their illness, which negatively influence their quality of life. Regardless of cancer type, unmanaged symptoms can lead to adverse downstream consequences. Patient Reported Outcome Measures (PROMs) can be used to inform patient care and lead to targeted symptom management but simply gathering this information does not improve outcomes for the patient. Patient generated information must be easy for the clinicians to access and interpret if it is to be used to inform care delivery in ambulatory oncology facilities. This pragmatic work responded to this need. One Canadian provincial ambulatory oncology jurisdiction implemented digital tracking of PROMs over time in the provincial Electronic Medical Record (EMR) to support full integration of PROMs into standard care workflows and processes. Due to an inability within the EMR for direct patient entry, a hybrid data-entry was designed where the patient completes a paper-based PROM in the waiting room, and after clinical review, a clinician documents this along with their clinical assessment in the EMR. Several digital dashboards were developed which report PROMs data at the micro (individual), meso (clinic) and macro (program) levels. Using PROMs routinely in these provincial practice settings has numerous benefits including enhanced patient-clinician communication, assisting with problem detection, management of symptoms, and improving outcomes for patients. There are over 60,000 unique patients represented in our PROMs database, and over 300,000 unique screening events captured. The PROMs data is now used at all levels of the provincial cancer jurisdiction to provide targeted person centred care (micro), to staff appropriately at a clinic or program level (meso), and for capacity planning for provincial programs (macro). A new provincial EMR is currently being implemented which has an associated patient portal. Based on the success of this work, integration of direct entry of PROMs by the patient prior to the appointment and an associated workflow for symptom management is underway in this jurisdiction.

## Background

Cancer patients experience distressing symptoms and concerns across the course of their illness which can negatively impact their quality of life [1] and lead to adverse downstream consequences [2, 3]. Understanding the combination of symptoms patients experience, how they have evolved, and how they impact quality of life, is essential to minimize suffering and provide targeted symptom management [2, 4]. Historically, most symptom data originated from clinical trials where severity and intensity were assigned based on clinician assessment [1, 5]; however, many patients now complete standardized, validated questionnaires to report their symptoms, concerns and well-being [6]. The information gathered on these questionnaires, commonly referred to as Patient Reported Outcome Measures (PROMs), can be used to inform patient care and symptom management [6]. Simply gathering this information does not improve patient outcomes [6]; patient generated information must be used strategically to inform care delivery in oncology clinics [7]. This pragmatic work responded to this need.

## Implementation of PROMs in Cancer Care Alberta (CCA)

Cancer Care Alberta (CCA) is the provincial cancer program in Alberta, Canada, where comprehensive PROMs data is collected alongside clinical and administrative data for most ambulatory cancer patients [8]. In 2012, CCA began routinely using a standardized PROM called “Putting Patients First” (PPF) to enhance symptom management [9, 10]. The PPF includes two standardized measures: the Revised Edmonton Symptom Assessment System (ESASr) [11, 12] and the Canadian Problem Checklist (CPC) [13]. These measures provide a comprehensive overview of common symptoms and concerns cancer patients experience, and align with national reporting requirements [11–13]. A Standard Operating Process (SOP) was developed to outline the expectation for repeated use of the PPF across the care trajectory, the method for collecting the patient reported information (paper-based), and the procedure for clinical review, assessment and response, including documentation [14].

In 2014, AHS endorsed the EuroQol Five Dimension Scale-5 Level (EQ5D-5L) [15] as the provincial health-related Quality of Life (HRQoL) PROM [16], allowing for comparisons of HRQoL across programs and location. The EQ5D-5L is currently used ad hoc in CCA for performance evaluation and economic analyses at the system level but planning is underway for its routine collection once a patient direct entry option is available provincially.

In 2015, funding was secured to develop visual tools that could digitally track PROMs over time, as clinicians voiced frustration with the paper-based PPF symptom screening process, which could not draw attention to worsening symptoms, or symptom clusters that contribute to suffering [17]. A hybrid data-entry process was designed where the PPF is completed in the waiting room and clinicians document the PROMs data along with their clinical assessment in the Electronic Medical Record (EMR) after its review. Currently in CCA, there are over 60,000 unique patients represented in our PROMs database, with over 300,000 digitally captured patient reports. Once this standard process was established, several digital dashboards were co-designed with IT/Analytics, clinicians and patient advisors to report PROMs data at the micro (individual), meso (clinic) and macro (program) levels.

### Micro: Trended Individual PROMs Dashboards

**Fig. 1:**
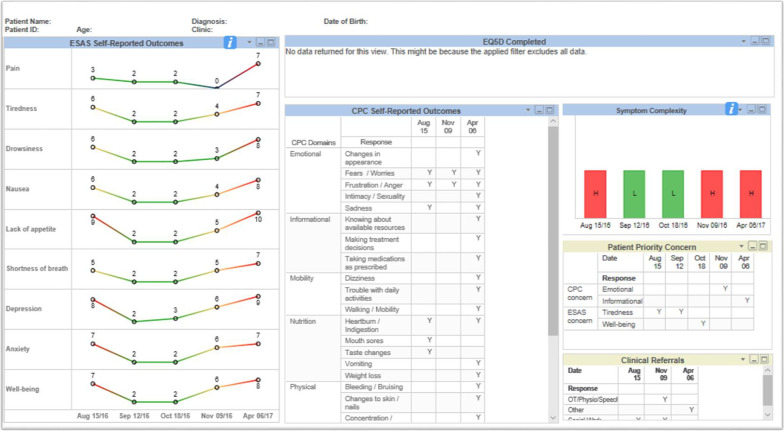
Trended Individual PROMs Dashboard

The initial dashboard prioritized for development by clinicians was the Trended Individual PROMs Dashboard (Trended Dashboard) for use at the individual patient level. The purpose of this dashboard was to help clinicians and patients understand how symptoms have changed over time. This displays data from the patient’s six most recent PPFs, the patient’s priority concern, and clinical actions taken in the encounter. Clinicians requested longitudinal trending on this dashboard to allow for clear visualization of how symptoms and concerns have changed over time (see Fig. 1).

Clinicians also requested visual cues to easily differentiate mild symptoms from moderate or severe which informed the color-coded trends. A patient rating of 0 (no symptom burden) is therefore represented in blue, 1–3 appears green, 4–6 appears yellow and scores between 7 and 10 (worst) appear red. Symptom complexity, which was added to the individual dashboard at a later stage, is also displayed (discussed in the next section). CPC items are reported in a corresponding summary table. Clinicians are able to select historical PPF data beyond the most recent six screening dates. Lastly, this dashboard indicates if, and when, a patient has also completed the EQ5D-5L [15]. While we worked quite extensively with CCA’s Enterprise Business Intelligence (EBI) team to develop this dashboard, ways in which the PROMs data could be reported and displayed were limited.

**Fig. 2:**
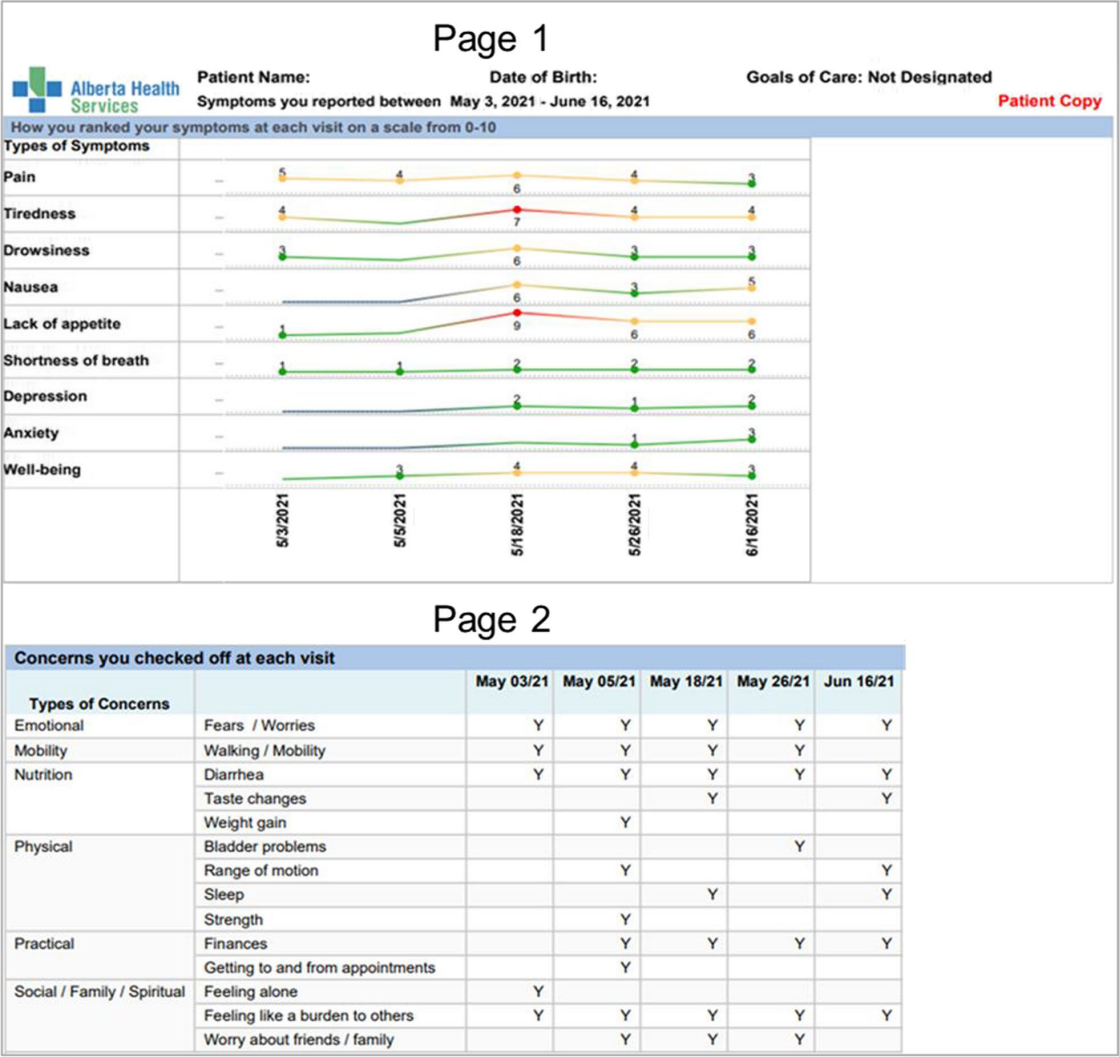
Patient-facing Symptom Tracking Report

A patient-facing version of the Trended Dashboard was also created, called the Symptom Tracking Report (STR). This simplified version, including only the elements reported by the patient, is printed out and given to patients upon clinic check-in, giving them time to review it independently and bring into their appointment to discuss with their care team (Fig. 2).

### Meso: PROMs Clinic List with symptom complexity score

As the first dashboard became integrated into practice, clinicians shared positive feedback about the utility, but indicated that looking up each patient’s dashboard was too time consuming, and requested a list of their patients from which each individual dashboard could be quickly accessed. Clinicians also requested a visual flag to identify patients struggling with a high number of symptoms or concerns at their last visit, so staff could anticipate which patients may need a more focused symptom assessment at their next appointment. This aligns with published research on barriers to using PROMs in routine practice including broad issues with the ease of interpretation and utilization of PROMs data in routine clinical care encounters^6^. Based on clinician input, the development of a symptom complexity algorithm and clinic list were the next two priority design areas.

**Fig. 3:**
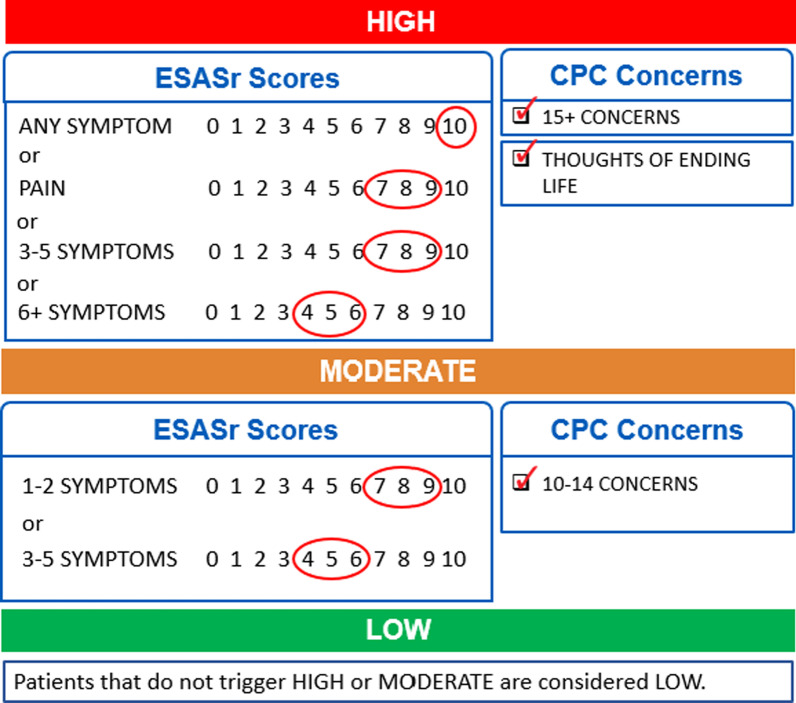
PROMs symptom complexity algorithm

The symptom complexity algorithm, which was again co-designed with clinicians, considers the severity of all ESASr symptoms and the number of CPC concerns reported on the PPF and assigns a symptom complexity score (mild/green, moderate/yellow or high/red) for the encounter. This clinician-facing summary score provides an alert to the clinical team so they can tailor their time allocation and care to best meet a patient’s needs. The final version of the symptom complexity algorithm is presented in Fig. 3 (validation of this algorithm is reported elsewhere) [18]. The symptom complexity score can also be used as a strategy to identify patients who are experiencing low symptom complexity, who could be triaged to other modes of care delivery outside of the in-person clinic (e.g. virtual appointment).

**Fig. 4:**
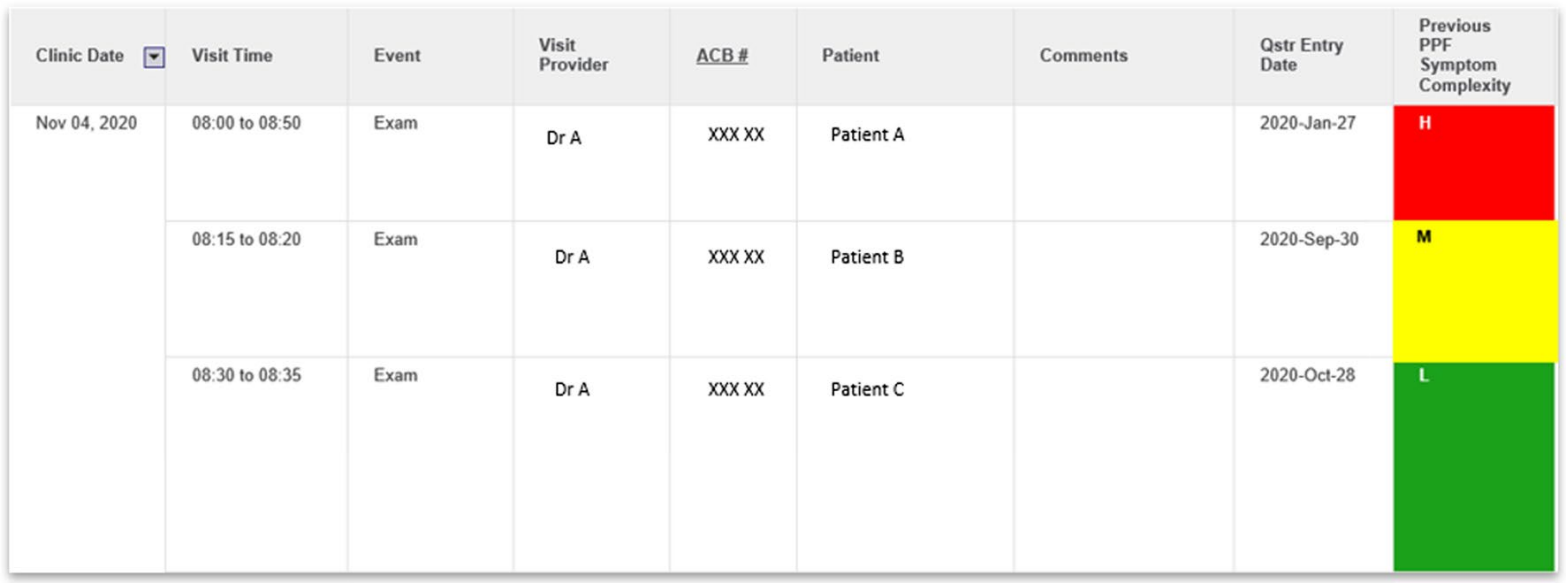
PROMs Clinic List

From there, the PROMs Clinic List with Symptom Complexity Score was developed (Fig. 4). Each patient on the list is hyperlinked to their own Trended Individual PROMs Dashboard, allowing clinicians a quick overview of the symptom burden of patients attending their clinic as well as access to the more detailed individual historical trends.

### Macro: PROMs Aggregate Dashboard

**Fig. 5:**
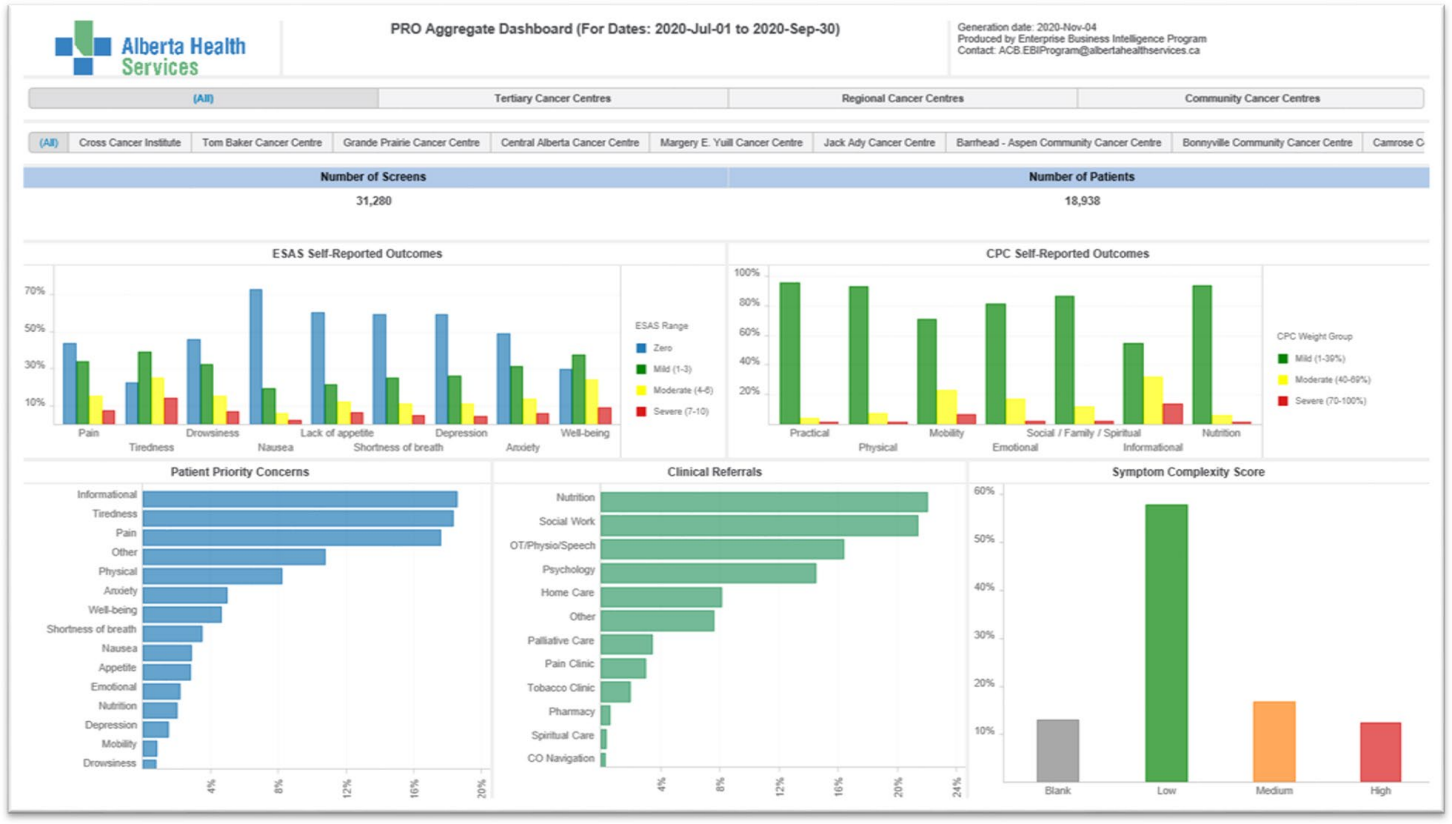
PROMs Aggregate Dashboard

The final dashboard developed was the PROMs Aggregate Dashboard which reports on symptoms, symptom complexity and referral activities for a variety of patient populations at the program level (Fig. 5). This dashboard is for clinical leaders and program administrators to identify populations that have high symptom complexity and referral data so staffing can be adjusted accordingly. This dashboard has also been used for comparisons across populations to determine those which may require embedded supportive care staff such as dieticians or psychologists. PROMs Aggregate Dashboard Data is reported nationally in the National Cancer System Performance Report created by the Canadian Partnership against Cancer (CPAC) [19], as many other cancer programs in Canada also use the ESASr as a PROM.

## Evaluation

As the primary goal in developing these dashboards was ultimately to improve patient communication and care processes, feedback was collected from 115 patients, all of whom had been given the STR in a recent visit, as a quality improvement (QI) initiative. Patients were surveyed about their use of the STR via telephone between January and April of 2019. A small number of staff from multiple CCA sites were surveyed regarding their use of the STR and PROMs Clinic List. The Alberta Innovates ARECCI Screening and Ethics Guideline Tools determined that this evaluation was QI with minimal risk [20]; full ethics approval was not required.

### Patient evaluation: results

Overall, patients were very positive about the STR and its use in the clinic. Specifically, 60.5% of patients agreed that the STR helped them talk to their health care team and 67% agreed that the report helped them understand how their symptoms have changed over time. The majority felt the report helped them recognize areas in which they could help themselves (60.9%), and areas in which they need additional support (60.5%). 77.4% of these patients rated their overall clinic experience, with the STR, positively. Patient comments including, “it is extremely useful the way it is,” and “I did not need help understanding the STR,” reinforce the positive impact of this resource on patient care.

### Staff evaluation: results

Survey results from 21 clinicians indicate that 76% felt comfortable using the STR as a communication tool with patients in the clinic and 67% appreciated the ability to easily gauge how patients’ symptoms changed over time. While 95% reported having the necessary skills and knowledge to use both the STR and the PROMs Clinic List, a lack of prep time prevented some staff members from routinely using the latter.

## Discussion

Interest is growing around using PROMs to monitor the progress of individual patients and inform their disease management, as research has shown PROMs to have numerous benefits including enhanced patient-clinician communication, assisting with problem detection, management of symptoms, and improving outcomes for patients [6]. The primary goal of capturing PROMs data is to improve outcomes for Albertans living with cancer. Encouragingly, based on patient feedback, the digital PROMs dashboards seem to be having a positive impact on improved patient care and experiences.

As CCA prepares to shift to a new EMR in May 2022, there is the potential to improve the function and usability of these dashboards. This EMR will have a patient direct-entry option for the collection of PROMs, so clinicians will no longer need to enter the PPF following the clinic visit. This will also enable patients to enter PROMs a few days prior to their appointment so that clinicians can view the most updated patient information in advance. Finally, there may be opportunities to expand the use of the PROMs dashboards beyond CCA, to pull in questionnaire data from other specialty health programs, as the new EMR will be implemented throughout Alberta’s health care system.

To overcome problems with interpretation, trending of an individual’s PROMs data over time allows clinicians and patients to see where their symptoms are getting worse and highlights the magnitude of this change. With regards to the time it takes to respond to PROMs, staff feedback suggests that additional strategies are needed to optimize time available in the clinic, as prep time for clinicians is often limited. A more robust staff evaluation about the use of these tools in the new EMR should be considered for the future, to ensure a comprehensive view of the utility of integrated PROMs dashboards is captured.

Lastly, using PROMs data to understand symptom complexity may allow for responsive staffing models to be created, as the provision of care can be tailored to provide the *right* level of intervention for the *right* patient, in the *right* setting, by the *right* provider, at the *right* time [21]. Work is underway in Alberta to examine the feasibility and economic impact of utilizing the symptom complexity algorithm as a core element within a new model of care delivery in CCA’s ambulatory cancer settings. PROMs are also being used to guide quality improvement for resource allocation, program development and as a component of economic analysis around value-based care.

## Conclusion

Cancer patients live with fluctuating symptoms across their cancer care trajectory. Routine collection and interpretation of PROMs data to monitor patients’ symptom complexity and inform targeted symptom management is becoming more common in ambulatory oncology settings [6, 8], but without attention to the barriers discussed, their impact in routine clinical care is minimal. This work provides a pragmatic approach to the visualization of PROMs and proposes the ability to improve symptom management capacity within existing cancer care resources. The use of the PROMs dashboards allows patients, clinicians and administrators to understand the symptoms, complexity, and concerns so that they can tailor their time allocation, staffing and resources to best meet the patient’s needs at the micro, meso and macro level.

## Data Availability

The datasets generated and/or analyzed during the current study are not publicly available due to confidential patient information but are available from the corresponding author on reasonable request.

## Abbreviations

CCA: Cancer Care Alberta
CPAC: Canadian Partnership Against Cancer
CPC: Canadian Problem Checklist
EBI: Enterprise Business Intelligence
EMR: Electronic Medical Record
EQ5D-5L: EuroQol Five Dimension Scale-5 Level
ESASr: Revised Edmonton Symptom Assessment System
HRQoL: Health-related Quality of Life
IT: Information technology
PPF: Putting Patients First
PROs: Patient Reported Outcomes
PROMs: Patient Reported Outcome Measures
QI: Quality improvement
SOP: Standard Operating Process
STR: Symptom Tracking Report
